# Reuse of gamma-ray irradiated textile wastewater: implications on the growth of *Capsicum frutescens* plant

**DOI:** 10.1016/j.heliyon.2022.e10009

**Published:** 2022-07-20

**Authors:** Md. Ariful Ahsan, M. Safiur Rahman, Md. Abdul Quaiyum Bhuiyan, Md. Saifur Rahaman, Mir Tamzid Rahman, Mubarak Ahmad Khan

**Affiliations:** a Isotope Hydrology Division, Institute of Nuclear Science and Technology, Atomic Energy Research Establishment, Bangladesh Atomic Energy Commission, Savar, Dhaka, Bangladesh; b Nuclear and Radiation Chemistry Division, Institute of Nuclear Science and Technology, Atomic Energy Research Establishment, Bangladesh Atomic Energy Commission, Savar, Dhaka, Bangladesh; c Chemistry Division, Atomic Energy Centre, Bangladesh Atomic Energy Commission, Savar, Dhaka, Bangladesh; d Department of Chemistry, Jahangirnagar University, Savar, Dhaka, 1342, Bangladesh; e Institute of Radiation and Polymer Technology, Atomic Energy Research Establishment, Bangladesh Atomic Energy Commission, Savar, Dhaka, Bangladesh; f Bangladesh Jute Mills Corporation, Bangladesh

**Keywords:** Textile wastewater treatment, Gamma radiation, Wastewater reuse, *Capsicum frutescens*, Plant growth

## Abstract

This investigation concentrates on the possibility of using gamma radiation for the decomposition of organic pollutants in textile wastewater and reuse as irrigation water. The wastewater sample was irradiated at four different absorbed doses of 3, 5, 8, and 10 kilo Gray (kGy). After irradiation at 8–10 kGy, physicochemical parameters, i.e., pH, turbidity, EC, total suspended solids (TSS) and total dissolved solids (TDS), have decreased sharply and approached to the expected value recommended by Department of Environment (DoE), Bangladesh. At 10 kGy absorbed dose, 59.0 % biological oxygen demand (BOD_5_) and 71.6 % chemical oxygen demand (COD) removal has been achieved, accelerating the enhancement in biodegradability index (BOD_5_/COD). Ammonium and total nitrogen have improved up to 87.0 % and 94.5 % after irradiation at 10 kGy doses. Subsequently, the treated textile wastewater samples were reused to grow *Capsicum frutescens* plants to inspect the fertility responses. When *Capsicum* plants were nourished by textile wastewater irradiated at 8–10 kGy, increased values were observed in the plant morphological parameters such as dry masses of the fruits (from 2.25 to 3.02 g), moisture content (from 91.35 to 92.62%), root length (from 13.21 to 16.56 cm), average plant height (from 2.42 to 4.07 cm/week), average number of leaves (from 14 to 16 nos./week), and total number of fruits (from 25 to 40 nos.) in comparison to those plants nourished by simply water and raw wastewater. The elemental analysis confirmed that negligible amounts of heavy metals were found in *Capsicum* fruits at higher absorbed doses. In contrast, helpful macro and micronutrients for plant production were raised to sufficient levels at 8–10 kGy, which can be the optimum doses for gamma irradiation to treat textile wastewater for maintaining sustainable water resources.

## Graphical abstract

**Figure:**
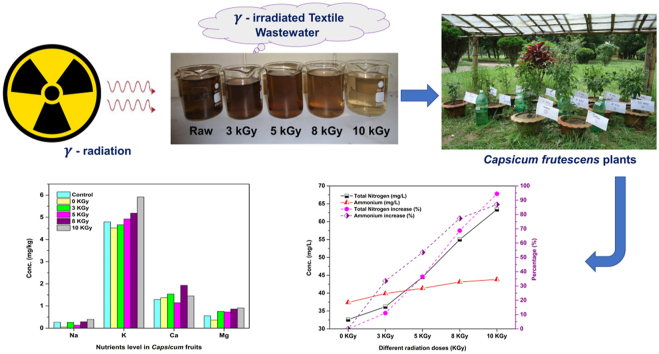

## 1 Introduction

Comprehensive industrialization and urbanization have produced the extreme discharge of wastewater into the water bodies worldwide, which have been influenced by the toxicity of industrial effluents. Continuous release of various hazardous elements from different industries has posed intense environmental pollution (Liu et al., 2016). Massive amounts of dyes have been produced to fulfil the annual requirements of various textile, leather, pharmaceuticals, paper, cosmetic and food industries. In the market, nearly 10,000 variations of synthetic dyes are found, whereas more than 700,000 tons are generated per annum all over the world (Das et al., 2021). It has been reported by the World Bank that around 17–20% of wastewater is produced from the textile finishing and dyeing treatment (Holkar et al., 2016). Textile industries are principal sources of discharging wastewaters which are extensively colored, highly alkaline, and contain refractory organic materials (Noman et al., 2013). Almost in all environments, industrial wastes act as a leading source of pollution, posing esthetic pollution, eutrophication, and perturbations in aquatic life because of their non-biodegradability, toxicity, carcinogenic, mutagenic natures (Amini et al., 2011). These pollutants lead to excessive suspended solids (SS), chemical oxygen demand (COD), color intensity, acidity, and basicity, exerting severe environmental problems (Şolpan et al., 2003). The existence of dyes and their byproducts in water can cause severe human health threats such as nausea, headache, skin irritation (Al-Mamun et al., 2019), hemorrhage, and can also cause extreme damage to the kidney, brain, liver, central nervous system and reproductive system (Akpor and Muchie, 2011).

As a remedy to this problem, industrial effluents require on-site treatment before discharging into the environment. Existing conventional methods like physical or chemical treatments cannot destroy the poisonous organic pollutants; instead, contaminants are transferred from an aqueous to a solid phase by the chemical coagulation method (Rabby, 2011). Thus, extensive sludge is generated from the coagulation process, creating secondary pollution if not properly handled (Al-Mamun et al., 2019). Adsorption techniques are lengthy, unable to generate a waste-free clear solution, and are not cost-effective (Islam et al., 2013). Reversely, biological processes are usually simple, environment friendly, have economic benefits (Al-Mamun et al., 2019), and are often used to remove toxic waste from textile wastewater (Kim et al., 2004). However, typical biological methods (e.g., activated sludge process) cannot rapidly eradicate these organic pollutants due to their large size, complex molecular structure, and chemical nature that converts them non-biodegradable in the environment (Kim et al., 2004). Advanced oxidation processes (AOPs) are also used to destroy wastewater dyes effectively, but their operation and maintenance costs are too high (Al-Mamun et al., 2019). In this case, the application of gamma radiation can be used as a remedy for textile wastewater treatment as this is more powerful, economical, and environmentally favorable (Changotra et al., 2019). Among many other ionizing radiations, the efficiency of gamma irradiation to degrade wastewater contaminants is broadly reported by Limam et al. (2018) and Wang et al. (2019). This treatment process gives some additional benefits such as irradiation performance is not affected by environmental conditions, no extra usage of chemicals, no residual or sludge generation (Hina et al., 2021), high penetration capability in various matrixes of water, and also unresponsive towards the insoluble solids that existed in wastewater (Changotra et al., 2019). The main advantages of radiation treatment over the conventional methods prescribe that this method is adaptable because of its easy management in a unit system, pollutants are destroyed by a rapid reaction mechanism (Hina et al., 2021) and its capability of simultaneous killing pathogenic microorganisms and destroying pollutants (Borrely et al., 1998).

Guin et al. (2014) investigated the cost competitiveness and treatment efficiencies between several advanced oxidation processes (AOPs) such as ozonolysis, photolysis, photocatalysis, and ionizing radiation radiolysis (gamma-ray) for the treatment of modified textile dye wastewater. The study found that ionizing radiation (gamma radiolysis) was a more effective treatment process for the mineralization of dye wastewater with a lower cost of 281 €/m^3^ of wastewater than ozonolysis (3421 €/m^3^ of wastewater) and photocatalysis (384 €/m^3^ of wastewater) treatment (Guin et al., 2014). In terms of environmental impact, gamma radiation is a promising technology capable of reducing the effects of biological and chemical pollution of industrial wastewater in the environment (Cabo Verde et al., 2015). The application of radiation technology for wastewater treatment is renowned internationally (IAEA, 2008). Increased use of ionizing radiation for treatment purposes is observed because of its well-developed compact design, larger capacity, reliability, and cost-effectiveness (Hossain et al., 2018).

Considering the international acceptability, crucial benefits over conventional methods, cost-effectiveness and reliability of the gamma irradiation treatment process, we have chosen to apply it in our study to treat textile wastewater. Nevertheless, in recent days, scientists worldwide have paid attention to reusing textile wastewater because of the increasing pollution load in the water bodies and groundwater depletion created by the textile industries (Bhuiyan et al., 2015). A few studies (Bhuiyan et al., 2014a, 2015; Parvin et al., 2015; Hina et al., 2021) have reported applying gamma-rays to treat textile effluents. However, unfortunately, very few studies have been conducted on treating industrial wastewater and recycling it on the growth and yield of the *Capsicum frutescens* plant. We have picked *Capsicum frutescens* in our study as it has been consumed as a spice all over the world. The fruits of *Capsicum frutescens* have a high content of ascorbic acid (Kumar and Tata, 2009), and leaves and fruit extracts have antifungal, antibacterial (Soumya and Nair, 2012) antioxidant and anti-inflammatory properties (Patricia et al., 2013).

The present study has been initiated to convert textile wastewater into resources in a sustainable manner. Successful completion of this study will create opportunities to reuse the wastewater as a fertilizer containing water, saving a massive cost for agricultural fertilizer and reducing freshwater withdrawals for irrigation. The specific objectives for this work are (i) application of irradiation technology to disintegrate the dye compounds and organic pollutants as well as to increase the biodegradability (BOD_5_/COD ratio) of the textile wastewater, (ii) to obtain the results of changes in physicochemical parameters and level of heavy metals of the irradiated textile wastewater, (iii) doses optimization of gamma irradiation for the treatment of the textile wastewater by applying different absorbed doses ranging from 3 kGy to 10 kGy, (iv) to explore the recycling suitability of gamma-irradiated textile wastewater by applying it into the vegetable species *Capsicum frutescens* and observing the growth rate and production effects of the plants and *Capsicum* fruits.

## 2 Material and methods

### 2.1 Sample collection and gamma irradiation

The composite textile wastewater samples were collected from the wastewater collection vessel from a knit dyeing textile industry, namely “Radial International Ltd.–Radiance Group” at Zirani Bazar, Kashimpur, Gazipur, Bangladesh. The samples were a composition of natural wastewater generated from different actions such as knitting, washing, and dyeing. The wastewater samples were gathered and sealed tightly in a 100 L clean and dry HDPE container and then sent for irradiation by gamma rays from the Cobalt-60 gamma source of the Institute of Radiation and Polymer Technology (IRPT), Atomic Energy Research Establishment, Savar, Dhaka, Bangladesh. The gamma radiation source was in batch irradiation mode, and the combined textile wastewater was irradiated at various absorbed doses 3, 5, 8 and 10 kilo Gray (kGy) at a dose rate of 13 kGy/h. An Amber Perpex dosimeter (type 3042F) has been used to measure the given dose values throughout the irradiation process.

### 2.2 Physicochemical analysis of raw and irradiated wastewater

The textile wastewater samples (both treated and untreated) were subjected to physical and chemical characterization, i.e., pH, turbidity, total suspended solids (TSS), total dissolved solids (TDS), electric conductivity (EC), dissolved oxygen (DO), biological oxygen demand (BOD_5_) and chemical oxygen demand (COD), to determine the optimum dose for decontamination. pH, TDS, and EC for irradiated and unirradiated samples were determined using a portable Multimeter (Model no. sension™ 156, HACH, USA, 2000) within 30 min from the sample collection time. The DO meter HQ40d from HACH, USA, was used to determine the DO values. BOD_5_ of the wastewater samples were analyzed by five days BOD_5_ test at 20 °C operating HACH DBR200 system following the standard procedures (APHA, 2017). A single beam UV-spectrophotometric system, model: DR/4000U, HACH International, Colorado, USA, with the help of reactor digestion method, was used to measure the COD values. The turbidity was measured by portable turbidity meter WTW TURB 350 IR. An oven-dried (30 min at 103–105 °C) fiber pad filter paper was weighed by analytical balance after cooling in desiccators for TSS measurement. Then 1000 mL samples were thoroughly shaken and filtered through the filter paper, followed by drying the filter paper in the oven (30 min at 103–105 °C), cooling in the desiccators, and then taking the dry weight of the materials (Bhuiyan et al., 2015). The total nitrogen and ammonium (NH_4_^+^) concentration of the treated and raw wastewater samples were also measured by the Kjeldahl and Kjeldahl distillation techniques (Mulvaney, 1996).

### 2.3 Experiments for the fertilizing effects by reusing of irradiated textile wastewater

*Capsicum frutescens* plants were irrigated three times a week by the irradiated and unirradiated wastewater to inspect the scope of reusing gamma-ray irradiated textile wastewater as irrigation water and its fertility impact. We prepared six types (twelve pots with twelve *Capsicum* plants in total – two plants per type) of *Capsicum* plant samples such as control (freshwater), 0 kGy (raw wastewater), 3, 5, 8 and 10 kGy irradiated textile wastewater. The plant samples were fed three times a week periodically by 250 mL volume of freshwater, raw wastewater and irradiated (3, 5, 8, 10 kGy) wastewater for respective types of plants. The control plant sample (irrigated by freshwater only) was used to compare the fertility effect of the raw and irradiated textile wastewater with the other plant samples. All the pots were kept under a transparent shed to avoid further mixing with rainwater. Garden soil with good moisture content had used to prepare the pots. Every week the plants were checked before assessing the plant height, the number of leaves and fruits to correlate the consequence of irradiated textile wastewater with the unirradiated and freshwater (control sample). For evaluating the growth and yield, plant height was estimated from margin of the pot to the peak of the central plant stem. For determining the number of leaves, every apparent leaf of each plant was considered, including the emerging tips of fresh leaves (Parvin et al., 2015). Every single apparent *Capsicum* fruit was also counted to measure the number of fruits of each plant. All the plants were harvested on the 64th day after implantation, and root lengths were measured. *Capsicum* fruits were collected from every plant, and the total weight of fruits (M_initial_) was calculated. Then the fruits were dehydrated in the microwave oven at 105 °C for 12 h (Mollah et al., 2009). After weight loss, the samples were collected from the oven, allowed to cool down in the desiccators, and after that weighed and recorded as dry mass (M_d_). Eq. (1) was used to figure out the moisture content (% MC) of the fruit samples (deMan et al., 2018).

(1) $$MC\text{ ​}\left(\text{\%}\right)\text{ ​}=\text{ ​}\left[\left(M_{initial}-M_{d}\right)\text{ ​/ ​}M_{initial}\right]\text{ ​× ​}100$$

Here, M_initial_ and M_d_ were the mass of the fruit samples before and after drying, respectively.

### 2.4 Elemental analysis of textile wastewater, soil, and capsicum fruits

A suitable volume of wastewater samples (both irradiated and unirradiated) was taken for the elemental analysis, filtered through Whatman 42 filter paper and then acidified by concentrated HNO_3_ until the pH ∼ 2. After that, 100 mL of samples with 5 mL concentrated HNO_3_ were taken and digested in a sealed chamber for 30 min. The end volume of samples was fixed up to 100 mL with distilled water (APHA, 2017). The harvested *Capsicum* fruit samples were washed with distilled water to clean the unwanted dust particles and soil. The samples were air-dried and collected into fresh polyethylene bags, sealed, and kept in the refrigerator. Subsequently, the solid samples (soil and fruits) were dried in the oven at 103–105 °C for 12 h and ground into fine dust (80 mesh size) utilizing a mortar for microwave digestion (Mollah et al., 2009). After that, 0.3 g of each plant and soil samples were weighed into the XP-1500 digestion vessel with 3 mL HNO_3_ (Conc.) acid for digestion in Microwave Accelerated Reaction System (MARS 5, CEM Corporation, USA). After completion of digestion, the concluding volume of the sample solutions was fixed up to 10 mL with distilled water. Digested wastewater and solid samples were then analyzed for metal concentrations by Atomic Absorption Spectrophotometer (Flame AAS, Varian AA240FS) and mercury (Hg) by cold vapor AAS, novAA350, Analytik Jena, Germany (Rahman et al., 2014).

## 3 Results and discussions

### 3.1 Radiolysis of textile wastewater by gamma radiation

Gamma radiation is one type of ionizing radiation with adequate energy to displace electrons from atoms and molecules, transforming them into electrically charged particles named ions (Selambakkannu et al., 2011). When gamma radiation is applied to the textile wastewater, radiolysis occurs in the water, producing excited and ionized water molecules with free electrons, highly reactive species (Bhuiyan et al., 2015). The application of gamma radiation is highly effective in aqueous solutions because the dye molecules found in the wastewater solution become degraded by the operation of primary products (•OH, e^-^_aq_. H^+^, •H, H_2_O_2_) produced by water radiolysis (Selambakkannu et al., 2011). Here hydroxyl radical (•OH) acts as an electrophile, a powerful oxidizing agent, whereas e^-^_aq_ radical, an effective reducing agent, behaves as a nucleophile with organic contaminants in the reaction (Buxton et al., 1988). Mainly the destruction of the conjugated system (N=N bonds) of the dye compounds occurs by the action of highly responsive hydroxyl radicals (•OH) (Sumartono, 2008). Moreover (•OH) radicals also attack the unsaturated bonds in benzene rings (Wojnarovits and Takacs, 2013), which finally prompts the disintegration of aromatic rings and generates acetaldehyde, carboxylic acids with other species into the solution (Wang and Chu, 2016). Because of the radiation effect, the longer organic chain degrades into shorter chains, which are adjoining to the major dye or azo groups (Nickelsen et al., 1992). Water radiolysis by ionizing radiation produces different chemical species as described in Eq. (2) (Spinks and Woods, 1990):

(2) $$H_{2}O\to \text{ ​}•H,\;•OH,\;{e^{-}}_{aq},\;•HO_{2},\;•H_{2}O_{2},\;H_{2},\;H^{+},\;OH^{-}$$

Degradation mechanism of azo dyes (Wojnarovits and Takacs, 2008) is shown below:

**Figure:**
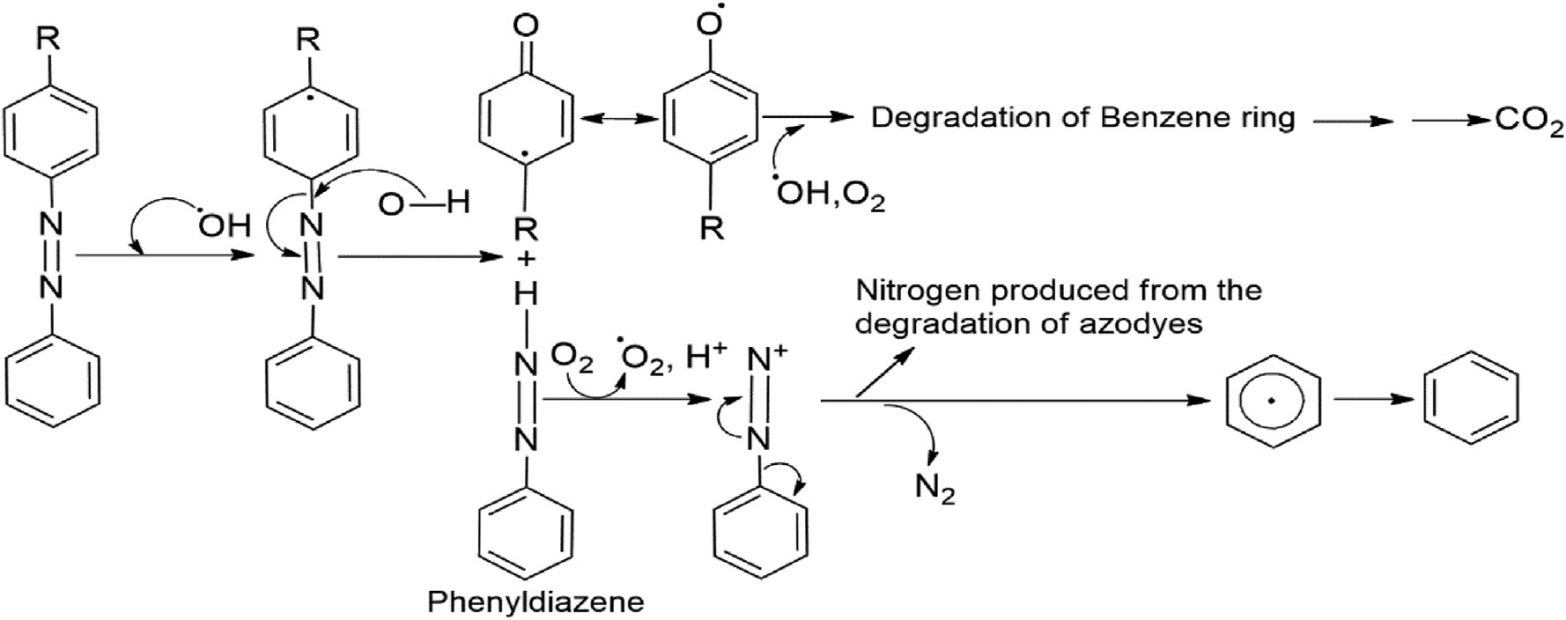

### 3.2 Effect of irradiation on physicochemical parameters in textile wastewater

The changes of physicochemical parameters for raw/unirradiated and irradiated wastewater are shown in Table 1. The main features of this textile wastewater were high pH, EC, TDS and TSS values with poor DO value. Similar values were also reported by Parvin et al. (2015) and comparatively lower values of pH, EC, TDS and TSS were found by Bhuiyan et al. (2015). The Pearson’s correlation analysis (Table 2) showed that pH has strong positive correlation with turbidity (*r* = 0.984, *p* = 0.002), EC (*r* = 0.0.992, *p* = 0.001), TDS (*r* = 0.990, *p* = 0.001), TSS (*r* = 0.985, *p* = 0.002), BOD_5_ (*r* = 0.997, *p* = 0.000), COD (*r* = 0.997, *p* = 0.002) and nitrate (*r* = 0.984, *p* = 0.002), while negative correlation was observed with DO (*r* = -0.996, *p* < 0.001), nitrogen (*r* = -0.971, *p* = 0.006) and ammonium (*r* = -0.992, *p* = 0.001). Therefore, due to increasing absorbed doses, reduced values of pH, turbidity, EC, TDS, TSS, BOD_5_, COD and NO_3_ were found, whereas DO, total nitrogen and ammonium showed increased values in the wastewater samples.

**Table 1:** Change of Physicochemical parameters of the irradiated and unirradiated samples of textile wastewater.

Parameters	Unit	Raw/Unirradiated wastewater			Standard for irrigation water (DoE, 1997)	Wastewater irradiated at different doses			
		This Study	Bhuiyan et al. (2015)	Parvin et al. (2015)		3 kGy	5 kGy	8 kGy	10 kGy
pH	-	10.48	8.3	10.33	6.0–9.0	9.72	9.18	8.64	8.19
Turbidity	FTU	167.22	-	161.65	-	153.83	139.29	118.56	116.68
EC	μS/cm	4010	2000	4140	1200	3640	2980	2160	1690
TDS	mg/L	3346	1050	3410	2100	2752	2460	1725	1540
TSS	mg/L	486	310	440	200	362	294	245	217
DO	mg/L	0.3	-	0.5	4.5–8.0	2.4	3.8	4.9	5.7

**Table 2:** Pearson correlation matrix of different physicochemical parameters, total nitrogen, ammonium and nitrate ions of raw and gamma irradiated textile wastewater.

	pH	Turbidity	EC	TDS	TSS	DO	BOD_5_	COD	Nitrogen	Ammonium	Nitrate
pH	1										
Turbidity	.984	1									
EC	.992	.991	1								
TDS	.990	.994	.992	1							
TSS	.985	.966	.959	.968	1						
DO	-.996	-.979	-.979	-.982	-.996	1					
BOD_5_	.997	.985	.997	.987	.970	-.987	1				
COD	.997	.991	.991	.996	.987	-.995	.992	1			
Nitrogen	-.971	-.970	-.991	-.973	-.916∗	.947∗	-.986	-.965	1		
Ammonium	-.992	-.985	-.978	-.982	-.996	.998	-.984	-.994	.944∗	1	
Nitrate	.978	.959∗	.987	.973	.927∗	-.955∗	.987	.969	-.993	-.945∗	1

Correlation is significant at the 0.01 level (2-tailed) for all numbers except those labelled with the symbol (∗).

This study revealed that the pH values in the textile wastewater were gradually decreased with the rise of the absorbed doses from 3 to 10 kGy. At an absorbed dose of 10 kGy, the pH value in wastewater was found to be 8.19, which was enough for reuse as irrigation water since it satisfied the standard range of irrigation water (DoE, 1997). Due to the action of gamma radiation, the highly reactive hydroxyl radicals (•OH) rapidly oxidizes the larger aromatic compounds in the wastewater and generate mono and dicarboxylic acids or carbonic acid (Wang and Chu, 2016). Eventually, that forms carbon dioxide by further oxidation and lowers the pH value of the wastewater (Paul et al., 2011).

The turbidity values for the textile wastewater samples were observed to reduce from 167.22-116.68 FTU. However, minimal variations of turbidity values were noticed for the doses of 8 kGy (118.56 FTU) and 10 kGy (116.68 FTU), with less visible differences too (Table 1). The reduction in turbidity is, in fact, for the decrease in suspended particulate matter. Still, the practical logic is the destruction of larger organic dye molecules and producing minor colorless organic species by applying gamma radiation (Soutsas et al., 2010). The dissolved oxygen (DO) of the unirradiated wastewater was found only 0.3 mg/L (Table 1). Nevertheless, after gamma irradiation, it had increased to the allowed value of 4.5–8 ppm for irrigation water (DoE, 1997). The DO value gradually increased from unirradiated to irradiated textile wastewater but at a slower rate at the end. It might have occurred because of the demolition of larger molecules, the decrease in turbidity (Table 1) of the wastewater samples, along with the existence of radiolysis products of water (O_2_, H_2_O_2_) due to gamma irradiation (Parvin et al., 2015).

At 10 kGy absorbed dose, the EC value became 1690 μS/cm, which was comparatively lesser than the EC value found in raw wastewater (4010 μS/cm) but not close to the standard value (1200 μS/cm) for irrigation water (DoE, 1997). However, higher absorbed doses were required to reduce the EC value because of ionized constitutes in the wastewater. EC has an approximate correlation with TDS, which was consistent with our Pearson’s correlation data (Table 2) having a strong positive correlation (*r* = 0.992, *p* < 0.005, *α* = 0.01). According to this study, with the increment of absorbed doses, EC values reduced significantly. A similar reduction tendency was also found for TDS (Table 1), which was 1540 mg/L at 10 kGy, lower than the recommended value of 2100 mg/L for irrigation quality of the water (DoE, 1997). The suspended solids content of the wastewater readily lowered after the gamma-ray irradiation (Table 1). The TSS value was 486 mg/L for unirradiated wastewater and 217 mg/L for 10 kGy absorbed dose, almost near to the standard TSS value (200 mg/L) for irrigation water as per DoE (1997). There are two probable causes of TDS and TSS reduction; the first is the deterioration of suspended dye molecules persuaded through the reaction with oxidative agents from hydrolysis of water (Somasiri et al., 2006). The second cause is the destruction of bigger organic molecules into tinier ones by radiation (Nickelsen et al., 1992).

**Figure 1:**
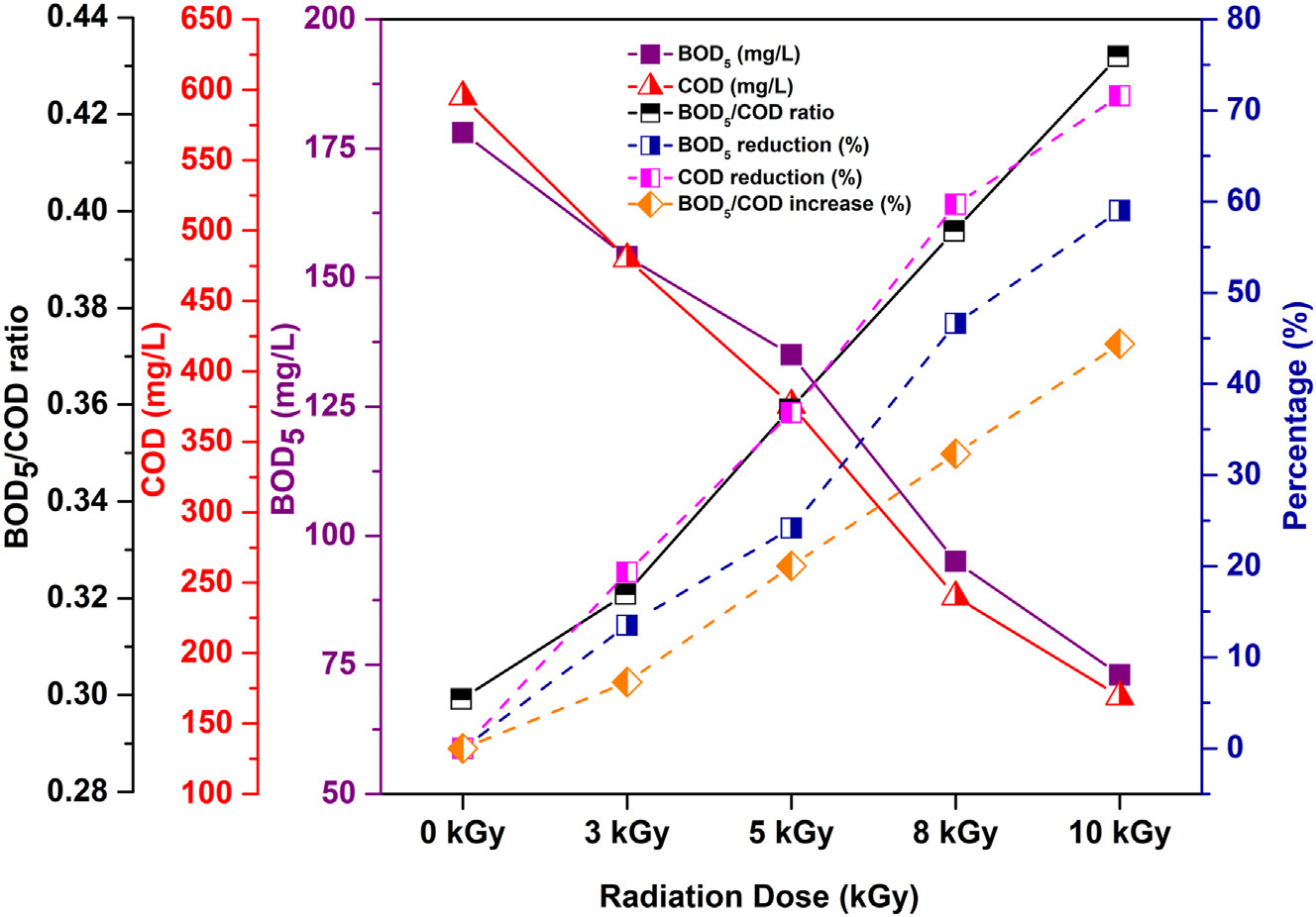
Changes of BOD_5_, COD, biodegradability index (BOD_5_/COD), BOD_5_ reduction (%), COD reduction (%) and BOD_5_/COD increase (%) in raw and irradiated textile wastewater.

In the case of biological oxygen demand (BOD_5_) and chemical oxygen demand (COD), a notable reduction in BOD_5_ and COD values of the wastewater is observed with increasing absorbed doses (Figure 1). The recommended standard limit of BOD_5_ and COD for irrigation water is 100 mg/L and 400 mg/L, respectively set by DoE (1997), duly achieved for the wastewater irradiated at 8–10 kGy in this study. The present study also revealed that at the highest absorbed dose of 10 kGy, 59.0 % and 71.6 % of BOD_5_ and COD removal were obtained. A strong positive correlation (*r* = 0.992, *p* < 0.005, *α* = 0.01) between BOD_5_ and COD was observed (Table 2). The highly reactive •OH radicals are produced by the radiolysis of wastewater reacting with suspended solid materials and degrading the organic contaminants (Selambakkannu et al., 2011). As a result, the degradation of these organic pollutants also reduces the bulk of biodegradable matters in wastewater, lowering BOD_5_ and COD values (Bhuiyan et al., 2015). The decline in COD values of the wastewater samples after radiation treatment could increase the biodegradability index (BOD_5_/COD) ratio, which is evident from Figure 1. However, the post-irradiation effect showed an elevation in BOD_5_/COD ratio from 0.3 to 0.43, and wastewater biodegradability index increased from 32.3 % to 44.4 % after irradiation at 8–10 kGy (Figure 1). For effective biological degradation of the wastewater biodegradability index (BOD_5_/COD) ratio value should be a minimum of 0.4 or higher (Al-Momani et al., 2002). In the present study, wastewater samples obtained biodegradability at 8–10 kGy absorbed doses because at these doses BOD_5_/COD ratio was 0.4–0.43 (Figure 1).

### 3.3 Effect of absorbed doses on total nitrogen and ammonium in textile wastewater

**Figure 2:**
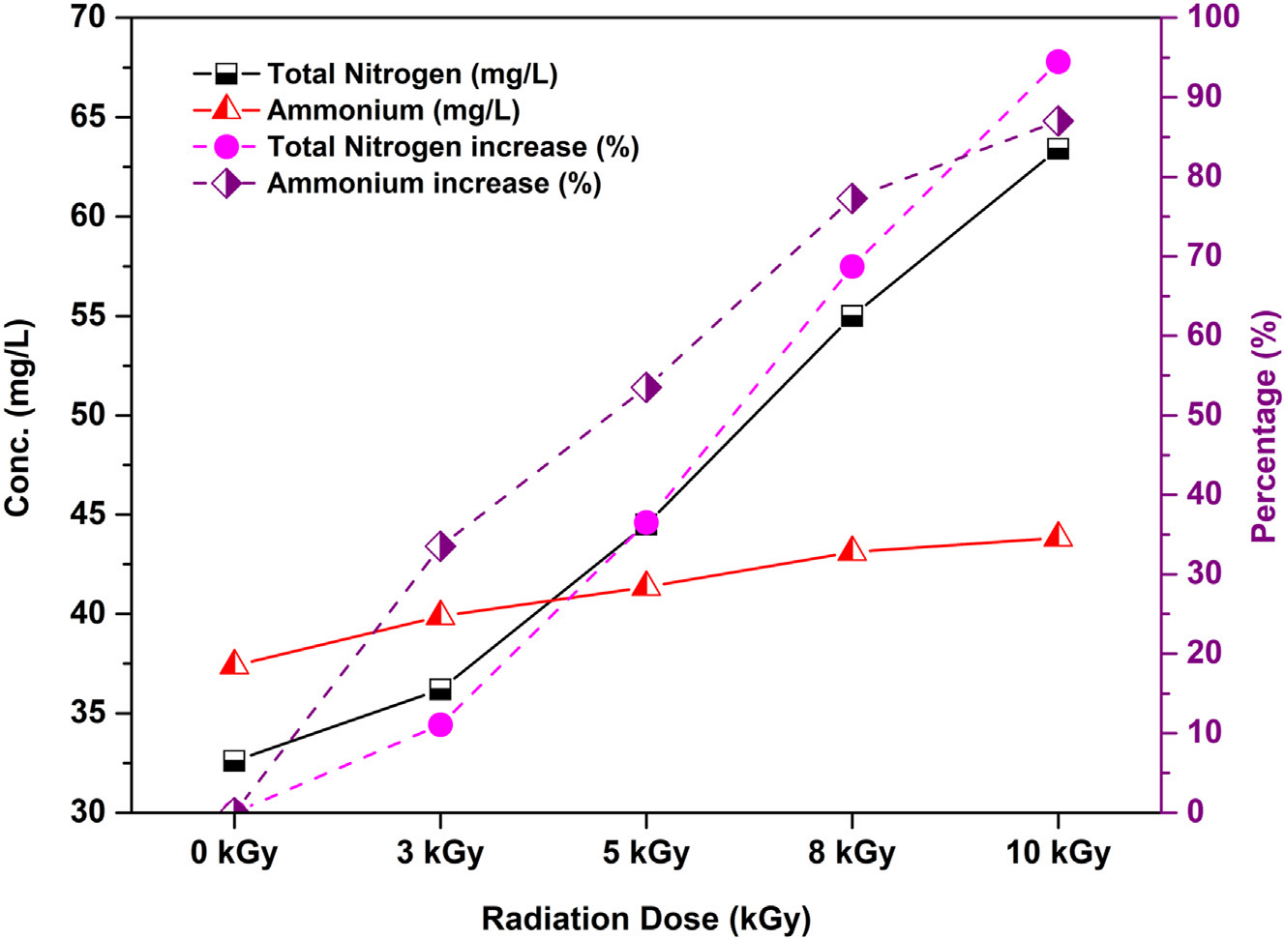
Changes of total nitrogen, ammonium level and their increase (%) in raw and irradiated textile wastewater.

This study revealed that the radiation treatment significantly improved the amount of total nitrogen (N) and ammonium (NH_4_^+^) contents in wastewater samples (Figure 2). The unirradiated wastewater contained only 32.6 mg/L and 18.5 mg/L of total nitrogen and ammonium, but when the wastewater was irradiated at 10 kGy, increased values were observed in total nitrogen (63.4 mg/L) and ammonium (34.6 mg/L). However, maximum 94.5% and 87.0% increases were found for total nitrogen and ammonium in the wastewater irradiated at highest absorbed dose of 10 kGy (Figure 2). The finding for applying absorbed doses on total nitrogen and ammonium in wastewater was completely reversed to changing pH value, which can be seen in the Pearson’s correlation data (Table 2). The Pearson’s correlation revealed a strong negative correlation between pH and total nitrogen (*r* = -0.971, *p* = 0.006, *α* = 0.01) and ammonium (*r* = -0.992, *p* < 0.005, *α* = 0.01). As a consequence of applying radiation, the stubborn portions of the azo dyes in wastewater have degraded efficiently, and thus nitrogen molecules appeared into the solution immediately after digestion (Nicklesen et al., 1992). Besides, gamma radiation converted the existing azo dyes in wastewater into amides, modified into ammonia by hydrolysis and then as ammonium ion, an important source of plant fertilizer (Bagyo et al., 1997).

### 3.4 Impact of absorbed doses on metals concentration in textile wastewater

This present study observed that the analyzed raw and irradiated wastewater samples carried a lower concentration of heavy metals (Table 3). The textile industry from where the wastewater samples were collected mainly consumes reactive and dispersed dyes for dyeing. Shore (2002) reported that metal complex groups are not found in dispersed dyes, and reactive dyes contain only 12–15 % of metal complex azo groups. Hence, it is expected to found a lower concentration of heavy metals in the studied wastewater samples. Heavy metals like Cr, Pb, Ni, and Cu are crucial because of their bio-accumulation solid capability, which could harm humans when introduced into the food cycle (Chiroma et al., 2014). Among the heavy metals, Cr, Co, Cu, Pb, Mn, Hg, Ni and Zn showed higher values in the wastewater samples irradiated at greater absorbed doses of 8–10 kGy compared to the unirradiated wastewater samples (Table 3). It might happen because the larger organic compounds in the wastewater contain metals in trapped or chelating form. When they get exposed to gamma irradiation, larger compounds break down into smaller chains, and ultimately the metals come out into the wastewater solution, increasing the concentration of metals (Parvin et al., 2015). According to the analytical report, arsenic (As) and cadmium (Cd) were less than the detection limit. However, the concentration of heavy metals in both unirradiated and irradiated wastewater samples was within the acceptable limits for using the wastewater as irrigation water (Ayers and Westcot, 1985; DoE, 1997; USEPA, 2012).

**Table 3:** Metal concentration (in mg/L) for raw and gamma irradiated textile wastewater.

Metal Name	Raw wastewater (0 kGy)	3 kGy	5 kGy	8 kGy	10 kGy	Standard for irrigation water^a^^,^^b^^,^^c^ (mg/L)		
						a	b	c
Arsenic (As)	<0.0003	<0.0003	<0.0003	<0.0003	<0.0003	0.2	0.1	0.1
Cadmium (Cd)	<0.004	<0.004	<0.004	<0.004	<0.004	0.05	0.01	0.01
Calcium (Ca)	0.2348	0.3562	0.3498	0.4028	0.3853	-	-	-
Chromium (Cr)	0.0791	0.0827	0.0848	0.0965	0.0950	1	0.1	0.55
Cobalt (Co)	<0.004	0.0077	0.0080	0.0086	0.0089	-	0.05	0.05
Copper (Cu)	0.0544	0.0630	0.0661	0.0863	0.0825	3	0.2	0.017
Iron (Fe)	0.0099	0.0217	0.0190	0.0364	0.0383	2	5	0.5
Lead (Pb)	0.0317	0.0402	0.0446	0.0505	0.0527	0.1	5	0.065
Magnesium (Mg)	0.0957	0.1883	0.1627	0.2199	0.2577	-	-	-
Manganese (Mn)	0.1605	0.2484	0.2347	0.2775	0.2914	5	0.2	0.2
Mercury (Hg)	0.0011	0.0012	0.0013	0.0015	0.0015	0.01	-	-
Nickel (Ni)	<0.004	0.0093	0.0103	0.0127	0.0133	1	0.2	1.4
Potassium (K)	0.3752	0.7229	0.9294	1.1403	1.2546	-	-	-
Sodium (Na)	0.1938	0.6570	0.5860	0.8274	0.9483	-	-	-
Zinc (Zn)	0.0120	0.0172	0.0166	0.0185	0.0188	10	2	0.2

a DoE (1997).

b USEPA (2012).

c Ayers and Westcot (1985).

Also, potassium (K), sodium (Na), calcium (Ca), magnesium (Mg), and iron (Fe) were found higher in the wastewater treated at absorbed doses of 8–10 kGy than in raw wastewater (Table 3), which could be helpful for plant growth as these are the vital micro and macronutrients for plants (Begum et al., 2011). In addition, heavy metals analysis of the soil used for plant cultivation was presented in Table 4. Almost all the heavy metal concentrations in the soil samples were within the maximum allowable limit (Chiroma et al., 2014; WHO, 2006). Moreover, the other metals like Na, Mg, Ca, K, and Fe, essential for plant growth, were present in an expected concentration that made the soil suitable for the experiments.

**Table 4:** Metal concentration (mg/kg) in soil for plant cultivation.

Metal Name	Concentration (mg/kg)	Maximum Allowable limit (mg/kg)	
		Chiroma et al. (2014)	WHO (2006)
Arsenic (As)	1.33	20	8
Cadmium (Cd)	0.55	3	4
Chromium (Cr)	1.33	100	-
Calcium (Ca)	0.55	-	-
Cobalt (Co)	42.14	50	-
Copper (Cu)	168.78	100	-
Iron (Fe)	6.67	50000	-
Lead (Pb)	18.26	100	84
Magnesium (Mg)	34.46	-	-
Manganese (Mn)	26.68	2000	-
Mercury (Hg)	82.68	-	-
Nickel (Ni)	126.22	50	107
Potassium (K)	<0.003	-	-
Sodium (Na)	39.64	-	-
Zinc (Zn)	142.84	300	-

### 3.5 Impacts of reused irradiated textile wastewater on capsicum plants and fruits

**Figure 3:**
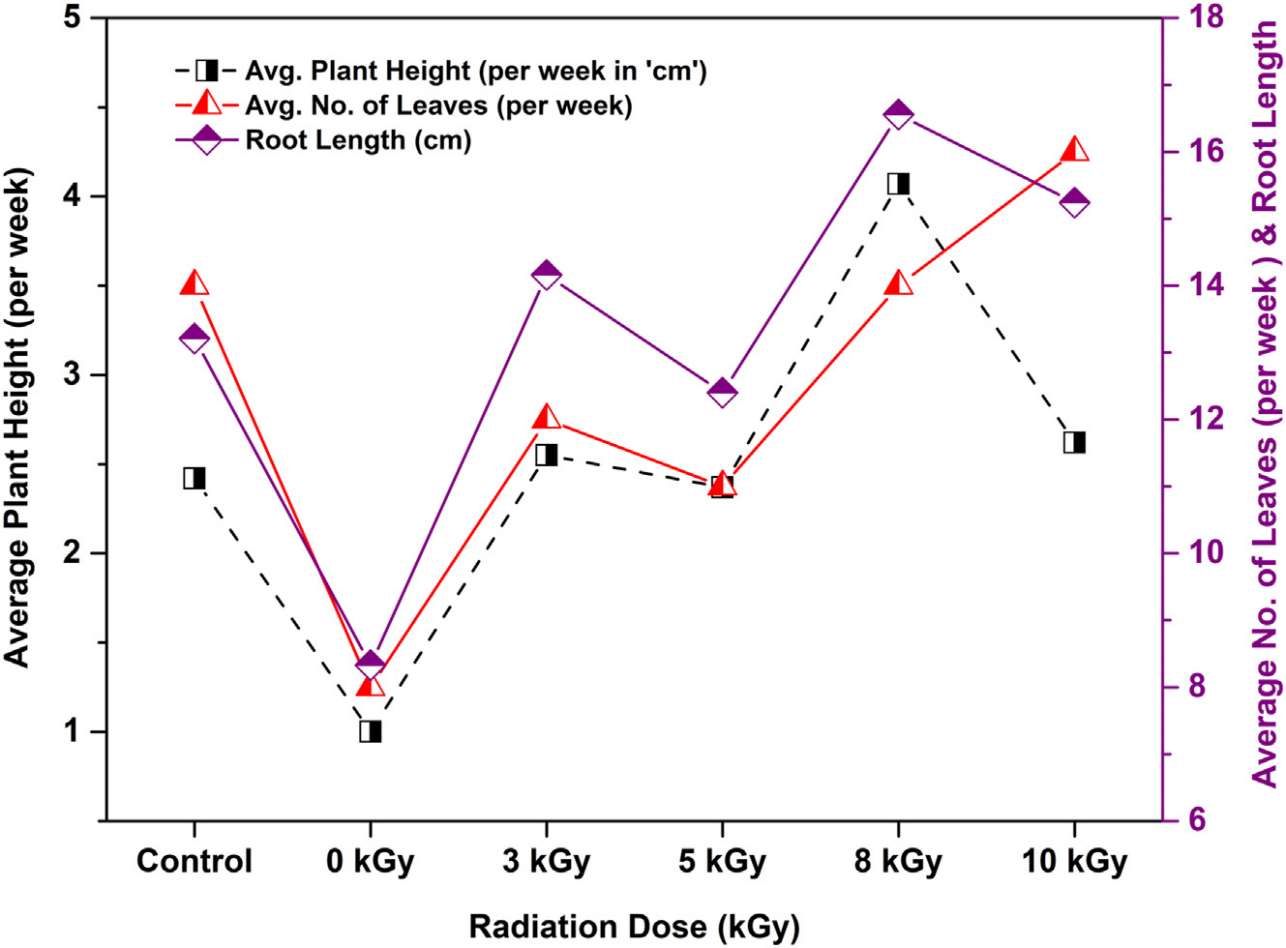
Increase in average plant height (cm/week), average number of leaves (per week) and root length (after harvesting) for control, unirradiated/raw and gamma ray irradiated *Capsicum* plants up to 64 days of the experiment.

In the present study, considerable changes were observed in plant morphologies for the *Capsicum* plants nourished by irradiated textile wastewater after 64 days of the experiment. Figure 3 shows the variation in plant growth parameters such as average plant height (‘cm’ per week), average number of leaves (per week), and root length up to 64 days (after harvesting) of *Capsicum frutescens* as a function of different absorbed doses on textile wastewater including the unirradiated and control samples. The highest average plant height (4.07 cm) and most average number of leaves (16 nos.) were found for the plants irrigated by 8 kGy and 10 kGy irradiated wastewater. Also, these morphological values of *Capsicum* plants nourished by only freshwater (control sample) and unirradiated wastewater were lower than the plant treated by gamma-ray irradiated (3–10 kGy) wastewater (Figure 3). Identical results were also observed in the case of root length for the *Capsicum* plants. As presented in Figure 3, the highest root length of 16.56 cm was found for the plants irrigated by 8 kGy, whereas 13.21 cm and 8.33 cm root lengths were found for the control sample and *Capsicum* plants treated with unirradiated textile wastewater, respectively. Notable growth in plant morphologies and root lengths were found for the plants irrigated by irradiated wastewater (Bhuiyan et al., 2015), because of the absorption of nutrients from the irradiated wastewater enriched with increased nutrients such as K, Na, Mg, and Zn (Begum et al., 2011).

**Figure 4:**
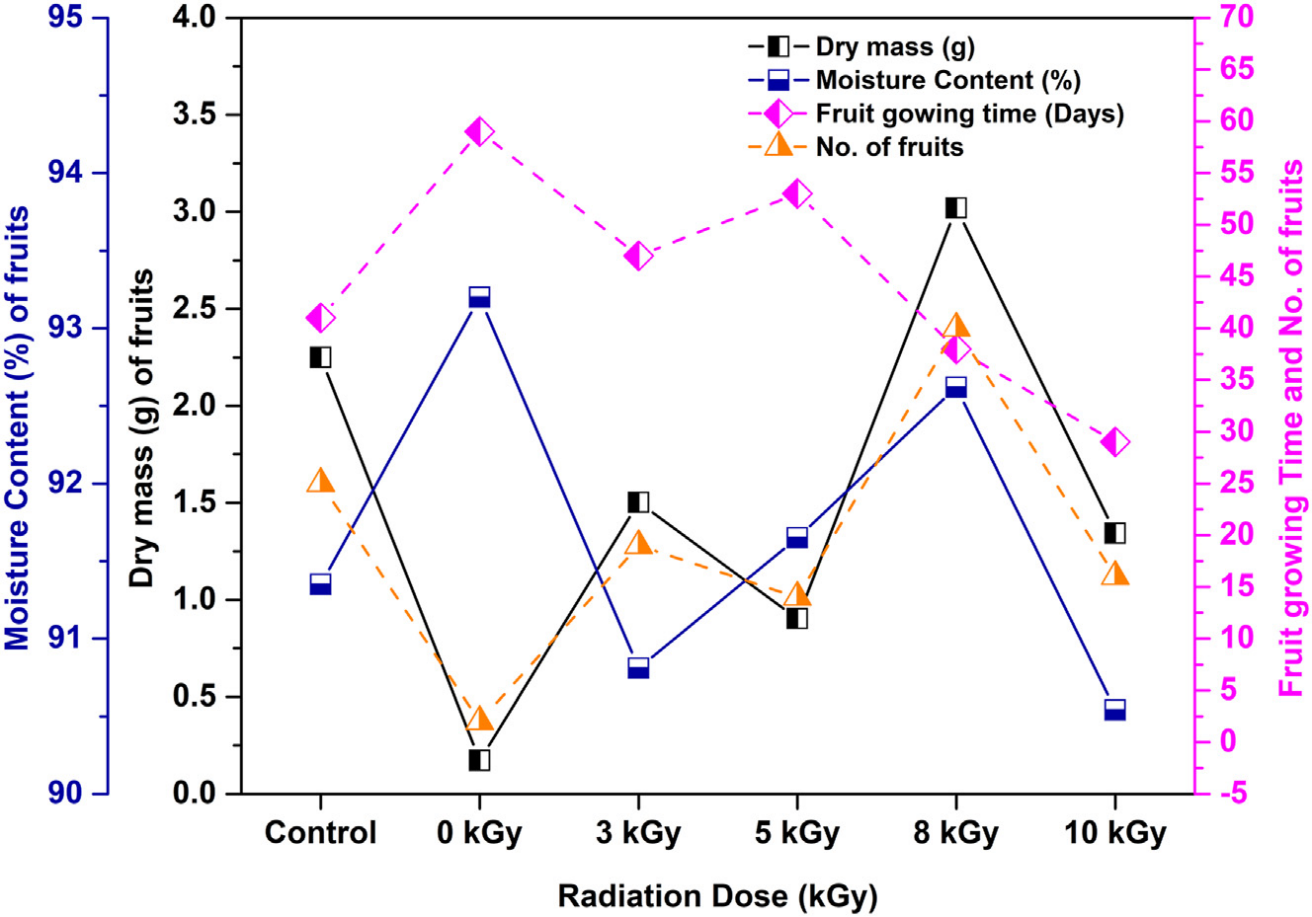
Variation in dry mass, moisture content (%), fruit growing time and no. of fruits for control, unirradiated/raw and gamma ray irradiated *Capsicum* plants after 64 days of the experiment.

Evident effects were also found on dry mass, moisture content (%), fruit growing time, and total number of fruits of *Capsicum* plants (Figure 4) after implementing gamma-ray irradiated textile wastewater. During the experimental time, *Capsicum* fruits grew after 29 days on the plant nourished by wastewater irradiated at 10 kGy. On the other hand, the plants fed with only water and raw wastewater, the fruits grew after 41 days and 59 days, respectively. Maximum 40 fruits and 3.02 g dry mass of these fruits were gained from the *Capsicum* plants treated by 8 kGy gamma-irradiated textile wastewater. The dry mass for the control sample was 2.25 g (total 25 fruits), and the plants treated with raw textile wastewater were 0.17 g (total two fruits only). According to Figure 4, the other plants treated with 3, 5, and 10 kGy gamma-ray irradiated textile wastewater showed a better result than the plants treated with only raw textile wastewater. Contrariwise, the highest moisture content (93.2%) was found for the fruits collected from the plants treated with raw wastewater, and 92.62% moisture content was found for 8 kGy fruit samples, which showed comparatively better performance among the irradiated and control fruit samples. Gamma irradiated textile wastewater possessed a higher concentration of nitrogen and ammonia (Bhuiyan et al., 2015), which ultimately influenced the increase in dry mass and moisture content of the *Capsicum* fruits (Parvin et al., 2015).

### 3.6 Metals concentration in capsicum fruits

**Figure 5:**
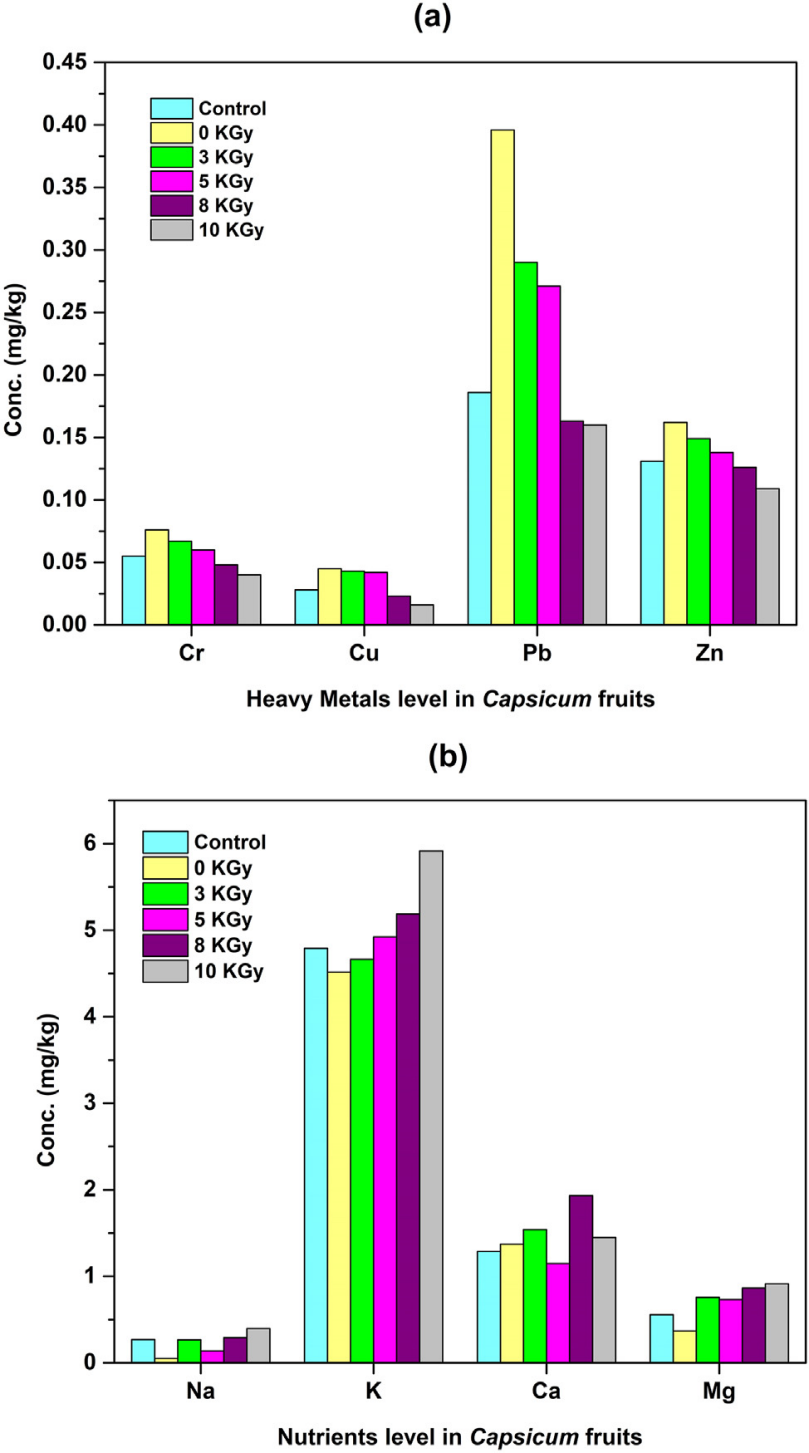
Concentration (mg/kg) of (a) heavy metals and (b) nutrients in *Capsicum* fruits collected from six types of *Capsicum* plant samples.

The analysis of *Capsicum* fruit samples for heavy metals concentration and the macro and micronutrients was done and presented in Figures 5a and 5b, respectively. The outcomes show that heavy metals concentration (Pb, Cr, Hg, Ni, Cu, and Zn) in *Capsicum* fruits decreased progressively as higher doses of treated wastewater were implemented (Figure 5a). The outcome indicates a distinguished translocation of these metals from the soil to the plant reproductive organs. At the highest absorbed dose of 10 kGy, Chromium (Cr) and lead (Pb) were found 0.04 mg/kg and 0.16 mg/kg for *Capsicum* fruit samples which were below the acceptable limit of 2.3 mg/kg and 0.3 mg/kg, respectively (Chiroma et al., 2014). At the same time, Nickel (Ni) and Mercury (Hg) in *Capsicum* fruit samples were found below the detection limit in elemental analysis, where 0.925 mg/kg Ni was found only in the fruit samples irradiated by raw wastewater. Copper (Cu) content was reported up to 0.016 mg/kg in the fruit samples nourished by 10 kGy gamma-ray irradiated textile wastewater (Figure 5a), which was exceedingly low compared to the highest permissible limit (73 mg/kg) of copper in vegetables (Chiroma et al., 2014). Zinc (Zn) concentration was 0.109 mg/kg in *Capsicum* fruit samples at 10 kGy, which was also insignificant against the maximum allowable limit of 100 mg/kg (Chiroma et al., 2014). Zn and Cu are essential nutrients for the plant that might be uptaken from the soil used for cultivation. Because Zn (142.84 mg/kg) and Cu (168.78 mg/kg) were found higher in the soil compared to the irradiated wastewater at 10 kGy (Zn = 0.0188 mg/L, Cu = 0.0825 mg/L).

Iron (Fe) is one of the essential metals for human health. However, the analytical result showed no iron metal consumed by the *Capsicum* fruits, not even in the fruit samples nourished with only water and raw wastewater. The concentration of different nutrients such as sodium (Na), potassium (K), magnesium (Mg), and calcium (Ca), which are crucial for plant growth, were found relatively more remarkable in the fruit samples of *Capsicum* plants cultivated by irradiated wastewater than in the plants grown by only water and raw wastewater (Figure 5b). Moreover, Na, K, and Mg had increased at a maximum level of 47.7%, 23.5% and 63.8%, respectively, for 10 kGy and the highest 50.0% rise in Ca concentration was found for 8 kGy among all the fruit samples. Different organic complexes were present in the raw wastewater (Bhuiyan et al., 2015), with nutrient elements as ligands that become degraded at higher doses of gamma radiation and released into the wastewater as free elements (Parvin et al., 2015). As a result, these free macro and micronutrients can be uptaken easily by the plants when applied to them (Parvin et al., 2015). Also, higher nutrient levels were found due to increased root lengths of the *Capsicum* plants irrigated by irradiated wastewater (Bhuiyan et al., 2015).

The treatment of textile wastewater by gamma irradiation has been accomplished. Recycling this treated wastewater by irrigating it into *Capsicum* plants also demonstrated a remarkable consequence at the optimized dose ranges. Several studies were performed to treat textile wastewater by ionizing radiation, but all those were carried out using self-prepared or simulated wastewater and failed to optimize the radiation doses. Our current study used textile wastewater from an industry that contained reactive and dispersive dyes comprised of less complex azo groups. The periodical discharge variation of wastewater from the industry and change in compositions of dye compounds were not considered in the present work. However, future studies should be intensified on conducting further research on the decontamination of extremely polluted industrial wastewater with various compositions of dye complexes by ionizing radiation and reusing treated wastewater in multiple sectors to justify the sustainability and commercial acceptability. In addition, for economic expansion government should step ahead to relax the legal regulations regarding the installation of radiation plants on a commercial basis so that the industrial sector can implement the irradiation technology comprehensively.

## 4 Conclusions

This present extensive investigation reveals that gamma radiation can efficiently break down the textile dyes and large organic contaminants in wastewater solutions which eventually reduce the pH, BOD_5_, COD, turbidity, EC, TDS, and TSS of textile wastewater. Significant improvements have been noticed in DO, ammonium, and total nitrogen content. The decline in BOD_5_ and COD values have influenced the increase in the biodegradability index (BOD_5_/COD >0.4) of irradiated wastewater. After implementing gamma-ray irradiated textile wastewater, the growth and production rate of the *Capsicum frutescens* have been reinforced in contrast to that of the plants cherished with unirradiated wastewater and only water. According to the elemental analysis report, the heavy metals exist in negligible amounts, but vital macro and micronutrients for plant development and human wellness such as Na, K, and Mg had raised at a satisfactory level of 47.7 %, 23.5 %, and 63.8 % for 10 kGy, whereas the highest 50.0 % increase in Ca concentration was found for 8 kGy fruit samples, indicating fascinating and fruitful results. The physicochemical features of irradiated wastewater, plants’ morphological characteristics, and *Capsicum* fruits production approach to a decent level at the absorbed doses of 8–10 kGy. So, 8–10 kGy absorbed doses are the optimized dose ranges for successful treatment of textile wastewater.

The outcome of this research will create a convenient way of recycling textile wastewater after being treated by gamma radiation efficiently in a sustainable manner. The reusing of irradiated textile wastewater as irrigation water in the agricultural field can save vast amounts of freshwater, prevent groundwater depletion, and reduce the extra expenses of fertilizers. Concurrently, irradiation treatment can mitigate potential environmental threats by eliminating harmful contaminants from industrial wastewater. As a whole, gamma radiation is an environmentally friendly alternative method for treating industrial wastewater, avoiding the use of toxic chemicals providing robust technology to the industries and a possible solution for sustainable management of vulnerable water resources.

## Declarations

### Author contribution statement

Md. Ariful Ahsan: Conceived and designed the experiments; Performed the experiments; Analyzed and interpreted the data; Contributed reagents, materials, analysis tools or data; Wrote the paper.

M. Safiur Rahman; Md. Saifur Rahaman: Analyzed and interpreted the data; Wrote the paper.

Md. Abdul Quaiyum Bhuiyan: Analyzed and interpreted the data; Contributed reagents, materials, analysis tools or data.

Mir Tamzid Rahman: Contributed reagents, materials, analysis tools or data.

Mubarak Ahmad Khan: Conceived and designed the experiments; Contributed reagents, materials, analysis tools or data.

### Funding statement

This research did not receive any specific grant from funding agencies in the public, commercial, or not-for-profit sectors.

### Data availability statement

Data will be made available on request.

### Declaration of interest’s statement

The authors declare no conflict of interest.

### Additional information

No additional information is available for this paper.
